# Entanglement entropy and edge modes in topological string theory. Part I. Generalized entropy for closed strings

**DOI:** 10.1007/JHEP10(2021)201

**Published:** 2021-10-26

**Authors:** William Donnelly, Yikun Jiang, Manki Kim, Gabriel Wong

**Affiliations:** 1 Perimeter Institute for Theoretical Physics, 31 Caroline St. N, Waterloo, ON N2L 2Y5 Canada; 2 Department of Physics, Cornell University, Ithaca, New York USA; 3 Department of Physics, Fudan University, Shanghai, China

**Keywords:** Gauge-gravity correspondence, Quantum Groups, Topological Field Theories, Topological Strings

## Abstract

Progress in identifying the bulk microstate interpretation of the Ryu-Takayanagi formula requires understanding how to define entanglement entropy in the bulk closed string theory. Unfortunately, entanglement and Hilbert space factorization remains poorly understood in string theory. As a toy model for AdS/CFT, we study the entanglement entropy of closed strings in the topological A-model in the context of Gopakumar-Vafa duality. We will present our results in two separate papers. In this work, we consider the bulk closed string theory on the resolved conifold and give a self-consistent factorization of the closed string Hilbert space using extended TQFT methods. We incorporate our factorization map into a Frobenius algebra describing the fusion and splitting of Calabi-Yau manifolds, and find string edge modes transforming under a *q*-deformed surface symmetry group. We define a string theory analogue of the Hartle-Hawking state and give a canonical calculation of its entanglement entropy from the reduced density matrix. Our result matches with the geometrical replica trick calculation on the resolved conifold, as well as a dual Chern-Simons theory calculation which will appear in our next paper [1]. We find a realization of the Susskind-Uglum proposal identifying the entanglement entropy of closed strings with the thermal entropy of open strings ending on entanglement branes. We also comment on the BPS microstate counting of the entanglement entropy. Finally we relate the nonlocal aspects of our factorization map to analogous phenomenon recently found in JT gravity.

## References

[1] Y. Jiang, M. Kim and G. Wong, *Entanglement entropy and edge modes in topological string theory. Part II. The dual gauge theory story*, *JHEP* **10** (2021) 202 [arXiv:2012.13397] [INSPIRE]. 10.1007/JHEP10(2021)201 PMC8550866 34725539

[2] Maldacena JM (1998). *The Large N limit of superconformal field theories and supergravity*. Adv. Theor. Math. Phys..

[3] Gubser SS, Klebanov IR, Polyakov AM (1998). *Gauge theory correlators from noncritical string theory*. Phys. Lett. B.

[4] Witten E (1998). *Anti-de Sitter space and holography*. Adv. Theor. Math. Phys..

[5] Ryu S, Takayanagi T (2006). *Holographic derivation of entanglement entropy from AdS/CFT*. Phys. Rev. Lett..

[6] Maldacena JM (2003). *Eternal black holes in anti-de Sitter*. JHEP.

[7] Faulkner T, Lewkowycz A, Maldacena J (2013). *Quantum corrections to holographic entanglement entropy*. JHEP.

[8] L.Y. Hung and G. Wong, *Entanglement branes and factorization in conformal field theory*, *Phys. Rev. D* **104** (2021) 026012 [arXiv:1912.11201] [INSPIRE].

[9] J. Lin, *Ryu-Takayanagi Area as an Entanglement Edge Term*, arXiv:1704.07763 [INSPIRE].

[10] Takayanagi T, Tamaoka K (2020). *Gravity Edges Modes and Hayward Term*. JHEP.

[11] Susskind L, Uglum J (1994). *Black hole entropy in canonical quantum gravity and superstring theory*. Phys. Rev. D.

[12] Donnelly W, Wong G (2017). *Entanglement branes in a two-dimensional string theory*. JHEP.

[13] Strominger A, Vafa C (1996). *Microscopic origin of the Bekenstein-Hawking entropy*. Phys. Lett. B.

[14] Tseytlin AA (1988). *Mobius Infinity Subtraction and Effective Action in σ Model Approach to Closed String Theory*. Phys. Lett. B.

[15] Dabholkar A (1995). *Strings on a cone and black hole entropy*. Nucl. Phys. B.

[16] He S, Numasawa T, Takayanagi T, Watanabe K (2015). *Notes on Entanglement Entropy in String Theory*. JHEP.

[17] Witten E (2019). *Open Strings On The Rindler Horizon*. JHEP.

[18] V. Balasubramanian and O. Parrikar, *Remarks on entanglement entropy in string theory*, *Phys. Rev. D* **97** (2018) 066025 [arXiv:1801.03517] [INSPIRE].

[19] Buividovich PV, Polikarpov MI (2008). *Entanglement entropy in gauge theories and the holographic principle for electric strings*. Phys. Lett. B.

[20] Donnelly W, Freidel L (2016). *Local subsystems in gauge theory and gravity*. JHEP.

[21] Donnelly W, Wall AC (2015). *Entanglement entropy of electromagnetic edge modes*. Phys. Rev. Lett..

[22] Donnelly W, Wall AC (2016). *Geometric entropy and edge modes of the electromagnetic field*. Phys. Rev. D.

[23] Kabat DN (1995). *Black hole entropy and entropy of entanglement*. Nucl. Phys. B.

[24] R. Gopakumar and C. Vafa, *M theory and topological strings. 1*, hep-th/9809187 [INSPIRE].

[25] R. Gopakumar and C. Vafa, *M theory and topological strings. 2*, hep-th/9812127 [INSPIRE].

[26] Gopakumar R, Vafa C (1999). *On the gauge theory/geometry correspondence*. Adv. Theor. Math. Phys..

[27] Gopakumar R, Vafa C (1998). *Topological gravity as large N topological gauge theory*. Adv. Theor. Math. Phys..

[28] Ooguri H, Vafa C (2000). *Knot invariants and topological strings*. Nucl. Phys. B.

[29] Aganagic M, Mariño M, Vafa C (2004). *All loop topological string amplitudes from Chern-Simons theory*. Commun. Math. Phys..

[30] Dedushenko M, Witten E (2016). *Some Details On The Gopakumar-Vafa and Ooguri-Vafa Formulas*. Adv. Theor. Math. Phys..

[31] Candelas P, de la Ossa XC (1990). *Comments on Conifolds*. Nucl. Phys. B.

[32] J. Bryan and R. Pandharipande, *Curves in Calabi-Yau 3 folds and topological quantum field theory*, math/0306316 [INSPIRE].

[33] Aganagic M, Ooguri H, Saulina N, Vafa C (2005). *Black holes, q-deformed* 2*d Yang-Mills, and non-perturbative topological strings*. Nucl. Phys. B.

[34] J. Bryan and R. Pandharipande, *The Local Gromov-Witten theory of curves*, *J. Am. Math. Soc.* **21** (2008) 101 [math/0411037] [INSPIRE].

[35] Lewkowycz A, Maldacena J (2014). *Exact results for the entanglement entropy and the energy radiated by a quark*. JHEP.

[36] Jensen K, Karch A (2013). *Holographic Dual of an Einstein-Podolsky-Rosen Pair has a Wormhole*. Phys. Rev. Lett..

[37] Jensen K, O’Bannon A (2013). *Holography, Entanglement Entropy, and Conformal Field Theories with Boundaries or Defects*. Phys. Rev. D.

[38] Gomis J, Okuda T (2007). *Wilson loops, geometric transitions and bubbling Calabi-Yau’s*. JHEP.

[39] Dymarsky A, Gubser SS, Guralnik Z, Maldacena JM (2006). *Calibrated surfaces and supersymmetric Wilson loops*. JHEP.

[40] Donnelly W, Timmerman S, Valdés-Meller N (2020). *Entanglement entropy and the large N expansion of two-dimensional Yang-Mills theory*. JHEP.

[41] Aguilera-Damia J, Correa DH, Fucito F, Giraldo-Rivera VI, Morales JF, Pando Zayas LA (2017). *Strings in Bubbling Geometries and Dual Wilson Loop Correlators*. JHEP.

[42] S.A. Gentle and M. Gutperle, *Entanglement entropy of Wilson loops: Holography and matrix models*, *Phys. Rev. D* **90** (2014) 066011 [arXiv:1407.5629] [INSPIRE].

[43] Hubeny VE, Pius R, Rangamani M (2019). *Topological string entanglement*. JHEP.

[44] G. ’t Hooft, *A Planar Diagram Theory for Strong Interactions*, *Nucl. Phys. B* **72** (1974) 461 [INSPIRE].

[45] Witten E (1995). *Chern-Simons gauge theory as a string theory*. Prog. Math..

[46] Witten E (1988). *Topological Sigma Models*. Commun. Math. Phys..

[47] Witten E (1998). *Mirror manifolds and topological field theory*. AMS/IP Stud. Adv. Math..

[48] Hartle JB, Hawking SW (1983). *Wave Function of the Universe*. Phys. Rev. D.

[49] Witten E (1986). *Noncommutative Geometry and String Field Theory*. Nucl. Phys. B.

[50] Witten E (1986). *Interacting Field Theory of Open Superstrings*. Nucl. Phys. B.

[51] Zwiebach B (1993). *Closed string field theory: Quantum action and the B-V master equation*. Nucl. Phys. B.

[52] Aganagic M, Klemm A, Mariño M, Vafa C (2005). *The Topological vertex*. Commun. Math. Phys..

[53] D. Maulik, A. Oblomkov, A. Okounkov and R. Pandharipande, *Gromov-Witten/Donaldson-Thomas correspondence for toric 3-folds*, arXiv:0809.3976 [INSPIRE].

[54] Donnelly W, Wong G (2019). *Entanglement branes, modular flow, and extended topological quantum field theory*. JHEP.

[55] R. Couvreur, J.L. Jacobsen and H. Saleur, *Entanglement in nonunitary quantum critical spin chains*, *Phys. Rev. Lett.* **119** (2017) 040601 [arXiv:1611.08506] [INSPIRE]. 10.1103/PhysRevLett.119.040601 29341751

[56] T. Quella, *Symmetry-protected topological phases beyond groups: The q-deformed Affleck-Kennedy-Lieb-Tasaki model*, *Phys. Rev. B* **102** (2020) 081120 [arXiv:2005.09072] [INSPIRE].

[57] D.L. Jafferis and D.K. Kolchmeyer, *Entanglement Entropy in Jackiw-Teitelboim Gravity*, arXiv:1911.10663 [INSPIRE].

[58] K. Hori et al., *Mirror symmetry*, vol. 1, American Mathematical Soc. (2003).

[59] A. Neitzke and C. Vafa, *Topological strings and their physical applications*, hep-th/0410178 [INSPIRE].

[60] Mariño M (2005). *Chern-Simons theory and topological strings*. Rev. Mod. Phys..

[61] Huang M-X (2019). *Recent Developments in Topological String Theory*. Sci. China Phys. Mech. Astron..

[62] M. Mariño, *Chern-Simons theory, matrix models, and topological strings*, vol. 131, Oxford University Press (2005) [DOI].

[63] M. Vonk, *A Mini-course on topological strings*, hep-th/0504147 [INSPIRE].

[64] Klemm A, Zaslow E (2001). *Local mirror symmetry at higher genus*. AMS/IP Stud. Adv. Math..

[65] Dijkgraaf R, Vafa C (2002). *Matrix models, topological strings, and supersymmetric gauge theories*. Nucl. Phys. B.

[66] Aganagic M, Dijkgraaf R, Klemm A, Mariño M, Vafa C (2006). *Topological strings and integrable hierarchies*. Commun. Math. Phys..

[67] C. Vafa, *Two dimensional Yang-Mills, black holes and topological strings*, hep-th/0406058 [INSPIRE].

[68] S.H. Katz and C.-C.M. Liu, *Enumerative geometry of stable maps with Lagrangian boundary conditions and multiple covers of the disc*, *Adv. Theor. Math. Phys.* **5** (2001) 1 [math/0103074] [INSPIRE].

[69] Gross DJ, Taylor W (1993). *Two-dimensional QCD is a string theory*. Nucl. Phys. B.

[70] Bershadsky M, Cecotti S, Ooguri H, Vafa C (1994). *Kodaira-Spencer theory of gravity and exact results for quantum string amplitudes*. Commun. Math. Phys..

[71] C. Vafa, *Brane/anti-brane systems and* U(*N*|*M*) *supergroup*, hep-th/0101218 [INSPIRE].

[72] W. Donnelly, *Decomposition of entanglement entropy in lattice gauge theory*, *Phys. Rev. D* **85** (2012) 085004 [arXiv:1109.0036] [INSPIRE].

[73] Gromov A, Santos RA (2014). *Entanglement Entropy in* 2*D Non-abelian Pure Gauge Theory*. Phys. Lett. B.

[74] Donnelly W (2014). *Entanglement entropy and nonabelian gauge symmetry*. Class. Quant. Grav..

[75] J. Kock, *Frobenius algebras and 2-d topological quantum field theories*, vol. 59, Cambridge University Press (2004).

[76] Atiyah MF (1988). *Topological quantum field theory*. Publ. Math. IHÉS.

[77] Lazaroiu CI (2001). *On the structure of open-closed topological field theory in two-dimensions*. Nucl. Phys. B.

[78] G.W. Moore and G. Segal, *D-branes and k-theory in* 2*D topological field theory*, hep-th/0609042 [INSPIRE].

[79] Runkel I, Szegedy L (2021). *Area-Dependent Quantum Field Theory*. Commun. Math. Phys..

[80] de Haro S, Ramgoolam S, Torrielli A (2007). *Large N expansion of q-deformed two-dimensional Yang-Mills theory and Hecke algebras*. Commun. Math. Phys..

[81] Witten E (1989). *Quantum Field Theory and the Jones Polynomial*. Commun. Math. Phys..

[82] Elitzur S, Moore GW, Schwimmer A, Seiberg N (1989). *Remarks on the Canonical Quantization of the Chern-Simons-Witten Theory*. Nucl. Phys. B.

[83] Guadagnini E, Martellini M, Mintchev M (1990). *Braids and Quantum Group Symmetry in Chern-Simons Theory*. Nucl. Phys. B.

[84] Seiberg N, Witten E (1999). *String theory and noncommutative geometry*. JHEP.

[85] A. Connes, *Noncommutative Geometry*, Academic Press (1995).

[86] Douglas MR, Nekrasov NA (2001). *Noncommutative field theory*. Rev. Mod. Phys..

[87] Woronowicz SL (1987). *Compact matrix pseudogroups*. Commun. Math. Phys..

[88] A. Klimyk and K. Schmudgen, *Quantum groups and their representations*, Springer (1997) [DOI].

[89] Kitaev A, Suh SJ (2019). *Statistical mechanics of a two-dimensional black hole*. JHEP.

[90] Blommaert A, Mertens TG, Verschelde H (2019). *Fine Structure of Jackiw-Teitelboim Quantum Gravity*. JHEP.

[91] E. Witten, (2 + 1)*-Dimensional Gravity as an Exactly Soluble System*, *Nucl. Phys. B* **311** (1988) 46 [INSPIRE].

[92] Teschner J (2001). *Liouville theory revisited*. Class. Quant. Grav..

[93] McGough L, Verlinde H (2013). *Bekenstein-Hawking Entropy as Topological Entanglement Entropy*. JHEP.

[94] M. Dupuis, L. Freidel, F. Girelli, A. Osumanu and J. Rennert, *On the origin of the quantum group symmetry in* 3*d quantum gravity*, arXiv:2006.10105 [INSPIRE].

[95] Mertens TG, Turiaci GJ (2021). *Liouville quantum gravity — holography, JT and matrices*. JHEP.

[96] Berkooz M, Isachenkov M, Narovlansky V, Torrents G (2019). *Towards a full solution of the large N double-scaled SYK model*. JHEP.

[97] J. Lin, *Entanglement entropy in Jackiw-Teitelboim Gravity*, arXiv:1807.06575 [INSPIRE].

[98] P. Saad, S.H. Shenker and D. Stanford, *JT gravity as a matrix integral*, arXiv:1903.11115 [INSPIRE].

[99] G. Penington, S.H. Shenker, D. Stanford and Z. Yang, *Replica wormholes and the black hole interior*, arXiv:1911.11977 [INSPIRE].

[100] Almheiri A, Hartman T, Maldacena J, Shaghoulian E, Tajdini A (2020). *Replica Wormholes and the Entropy of Hawking Radiation*. JHEP.

[101] R. Dijkgraaf, R. Gopakumar, H. Ooguri and C. Vafa, *Baby universes in string theory*, *Phys. Rev. D* **73** (2006) 066002 [hep-th/0504221] [INSPIRE].

[102] Aganagic M, Ooguri H, Okuda T (2007). *Quantum Entanglement of Baby Universes*. Nucl. Phys. B.

[103] Coleman SR (1988). *Black Holes as Red Herrings: Topological Fluctuations and the Loss of Quantum Coherence*. Nucl. Phys. B.

[104] Giddings SB, Strominger A (1988). *Loss of Incoherence and Determination of Coupling Constants in Quantum Gravity*. Nucl. Phys. B.

[105] Giddings SB, Strominger A (1989). *Baby Universes, Third Quantization and the Cosmological Constant*. Nucl. Phys. B.

[106] D.L. Jafferis, *Bulk reconstruction and the Hartle-Hawking wavefunction*, arXiv:1703.01519 [INSPIRE].

[107] Marolf D, Maxfield H (2020). *Transcending the ensemble: baby universes, spacetime wormholes, and the order and disorder of black hole information*. JHEP.

[108] J. McNamara and C. Vafa, *Baby Universes, Holography, and the Swampland*, arXiv:2004.06738 [INSPIRE].

[109] J. McNamara and C. Vafa, *Cobordism Classes and the Swampland*, arXiv:1909.10355 [INSPIRE].

[110] Vafa C (1996). *Gas of D-branes and Hagedorn density of BPS states*. Nucl. Phys. B.

[111] Candelas P, De La Ossa XC, Green PS, Parkes L (1998). *A Pair of Calabi-Yau manifolds as an exactly soluble superconformal theory*. AMS/IP Stud. Adv. Math..

